# Immunostimulative Activity of Low Molecular Weight Chitosans in RAW264.7 Macrophages

**DOI:** 10.3390/md13106210

**Published:** 2015-09-30

**Authors:** Ning Wu, Zheng-Shun Wen, Xing-Wei Xiang, Yan-Na Huang, Yang Gao, You-Le Qu

**Affiliations:** 1 Zhejiang Provincial Engineering Technology Research Center of Marine Biomedical Products, Food and Pharmacy College, Zhejiang Ocean University, Zhoushan 316000, China; E-Mail: wun@dxy.cn; 2 Zhejiang Marine Development Research Institute, Zhoushan 316000, China; E-Mail: xxw11086@126.com; 3 College of Animal Science and Technology, Guangxi University, Nanning 530004, China; E-Mail: huangyn@gxu.edu.cn; 4 School of Fishery, Zhejiang Ocean University, Zhoushan, 316000, China; E-Mail: avgy1982@hotmail.com

**Keywords:** immunostimulative activity, low molecular weight chitosans, cytokines, macrophages

## Abstract

Chitosan and its derivatives such as low molecular weight chitosans (LMWCs) have been reported to exert many biological activities, such as antioxidant and antitumor effects. However, complex and molecular weight dependent effects of chitosan remain controversial and the mechanisms that mediate these complex effects are still poorly defined. This study was carried out to investigate the immunostimulative effect of different molecular weight chitosan in RAW264.7 macrophages. Our data suggested that two LMWCs (molecular weight of 3 kDa and 50 kDa) both possessed immunostimulative activity, which was dependent on dose and, at the higher doses, also on the molecular weight. LMWCs could significantly enhance the the pinocytic activity, and induce the production of tumor necrosis factor α (TNF-α), interleukin 6 (IL-6), interferon-γ (IFN-γ), nitric oxide (NO) and inducible nitric oxide synthase (iNOS) in a molecular weight and concentration-dependent manner. LMWCs were further showed to promote the expression of the genes including iNOS, TNF-α. Taken together, our findings suggested that LMWCs elicited significantly immunomodulatory response through up-regulating mRNA expression of proinflammatory cytokines and activated RAW264.7 macrophage in a molecular weight- and concentration-dependent manner.

## 1. Introduction

Low immune function of an organism may not only result in the generation and development of a tumor, but may also be one of the most important factors that prevent the tumor patient’s recovery. Immunomodulation through natural or synthetic substances may be considered an alternative for the prevention and cure of diseases [1]. Macrophages play a significant role in the host defense mechanism. When activated, they activate phagocytic activity, produce and release reactive oxygen species (ROS) and nitric oxide (NO) in response to stimulation with various agents, and can inhibit the growth of a wide variety of tumor cells and microorganisms [2,3]. Macrophages also secrete cytokines and chemokines, such as tumor necrosis factor (TNF-α), interleukin-1 (IL-1) and interferon-γ (IFN-γ). Moreover, the immunomodulatory activity not only involves effects on cell proliferation and differentiation but also on macrophage activation [4]. Macrophages occupy a unique niche in the immune system, in that they can not only initiate innate immune response, but can also be effector cells that contribute to fight infection and inflammation. Following activation, macrophages can induce expression of accessory and costimulatory molecules that promote sustained stimulatory interactions with T cells and the generation of adaptive immunity. Indeed, the basic mechanisms of the immunostimulatory, anti-tumor, bactericidal and other therapeutic effects of polysaccharides are thought to occur via activation of immune cells resulting in the induction of immune response. Macrophages were thought to be the important target cells of some antitumor and immunomodulatory drug [5].

Polysaccharides obtained from natural sources represent a structurally diverse class of macromolecules, and are known to affect a variety of biological responses, especially the immune response. Chitosan is an abundant, natural linear polysaccharide derived by the deacetylation of chitin from crustaceans, insects and fungi. Chitosan is non-toxic (Lethal Dose _50_ > 16 g/kg), biodegradable, non-immunogenic and can be manufactured reproducibly in accordance with GMP guidelines [6]. Chitosan is a widely used biomaterial with an established safety profile in humans. It is used an experiment mucosal adjuvant [7,8,9] and vaccine adjuvant in mice [10]. Recently, chitosan has received considerable attention for its commercial applications in the biomedical, food, and chemical industries. The biomedical applications of various forms of chitosan have long been studied. Chitosan derived from chitin is of high molecular weight with poor solubility and, ultimately, unsatisfactory its therapeutic potential. To address these poor physicochemical properties, more active forms, like low molecular weight chitosans (LMWCs) have been generated [11]. In addition, chitosans derivatives seem to activate macrophage secreting cytokines such as interferon-γ (IFN-γ), interleukins [12]. LMWCs, which are more effectively absorbed in the body than high molecular weight chitosan, suitable narrow molecular weight distribution and non-toxicity, could be applied most promisingly to pharmaceutical materials. However, there was no clear information describing the relationship between molecule weight properties and immunostimulative activity of LMWCs. Therefore, we hypothesized that LMWCs may have a potential to augment the immunomodulatory activity in molecular-weight-dependent manner. RAW264.7 cells are commonly accepted as a tool to investigate the molecular mechanisms of macrophages involved in regulating immunity. Herein, we investigated the immunomodulatory activity of LMWCs (3 kDa and 50 kDa) in murine macrophages RAW264.7 cells and to develop a mechanistic understanding on size-dependent effects of chitosan on innate immune responses. The current experiments were designed to investigate the immunomodulatory effects of LMWCs on RAW264.7 macrophages by determining the effect on pinocytic activity, the production of nitric oxide and cytokines and their genes expression.

## 2. Results

### 2.1. Effects of LMWCs on the Cell Viability of RAW264.7 Macrophage

**Figure 1:**
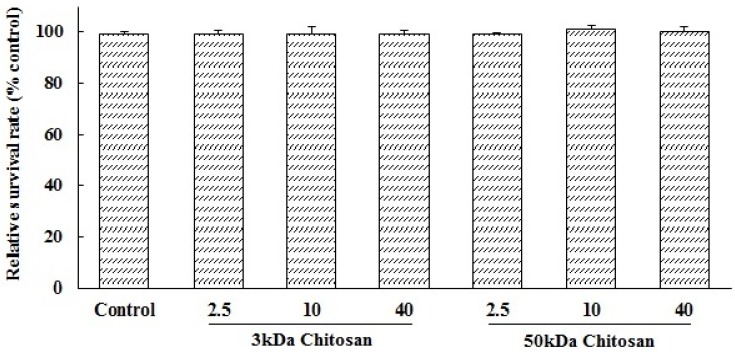
Effects of low molecular weight chitosans (LMWCs) on the cell viability of RAW264.7 macrophage. Each cell population (2 × 10^4^ cells/well) was treated with LMWCs (3 kDa and 50 kDa) at the indicated concentrations of 2.5, 10 and 40 μg/mL for 24 h, respectively. Values are means ± SD (*n* = 3). Bars with no letters are not statistically different (*p* > 0.05).

LMWCs (3 kDa and 50 kDa) were investigated for their immunostimulative activity in RAW264.7 macrophages. To evaluate possible cytotoxicities of LMWCs on RAW264.7 macrophages, LMWCs at the indicated concentrations of 2.5, 10 and 40 μg/mL were cultured with cells for 24 h, respectively. The results showed that RAW264.7 macrophages viability was not significantly (*p* > 0.05) influenced by LMWCs at the indicated concentrations of 2.5, 10 and 40 μg/mL (Figure 1). Therefore, LMWCs at the indicated concentrations were selected to conduct assay of immunomodulatory activity.

### 2.2. Effects of LMWCs on Pinocytic Activity

**Figure 2:**
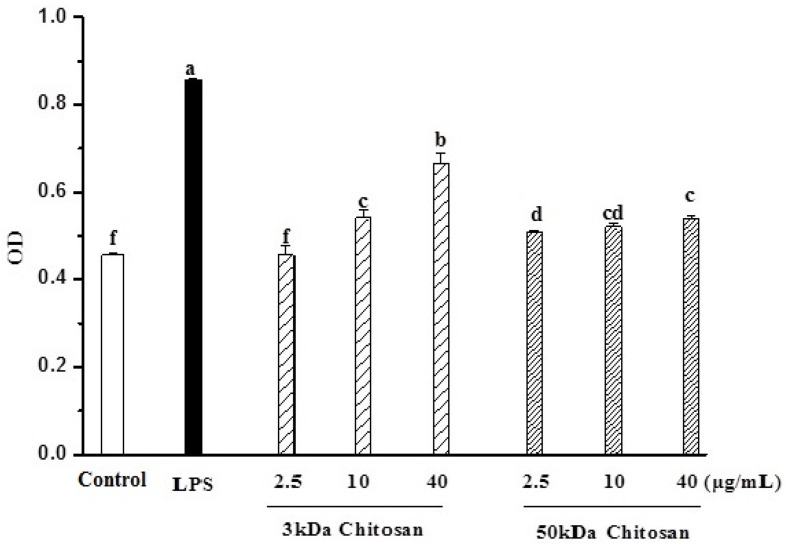
Effects of LMWCs on the pinocytic activity of RAW264.7 macrophage. Each cell population (1 × 10^4^ cells/well) was treated with LMWCs (3 kDa and 50 kDa) at the indicated concentrations of 2.5, 10 and 40 μg/mL for 24 h, respectively. Values are means ± SD (*n* = 3). Bars with different letters (a, b, c, d, e, f) are statistically different (*p* < 0.05).

The effect of LMWCs on the pinocytic activity of RAW264.7 cells was examined by the uptake of neutral red. As shown in Figure 2, LMWCs (3 kDa and 50 kDa) significantly enhanced the pinocytic activity of RAW264.7 cells in a dose-dependent manner (*p* < 0.05). Meanwhile, we also found that 3 kDa chitosan at doses (10, 40 μg/mL) significantly promoted the pinocytic activity compared with that at same dose of 50 kDa chitosan, suggesting that LMWCs significantly induced the pinocytic activity of macrophages dependent on its size and dose.

### 2.3. Effect of LMWCs on Macrophage Cytokines Production

**Figure 3:**
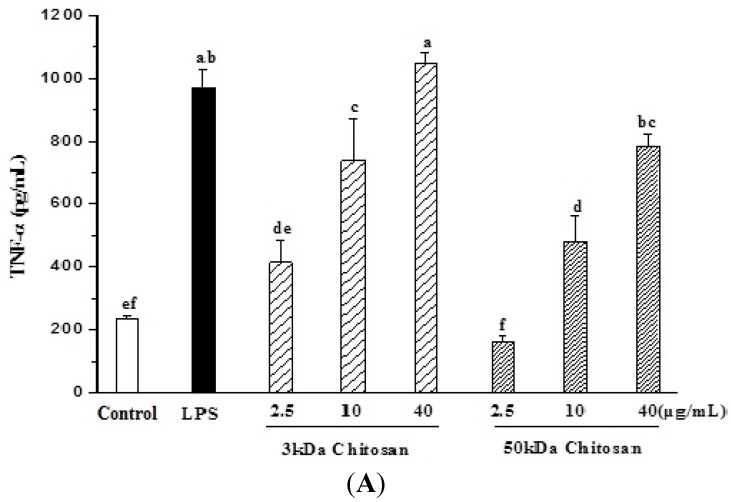

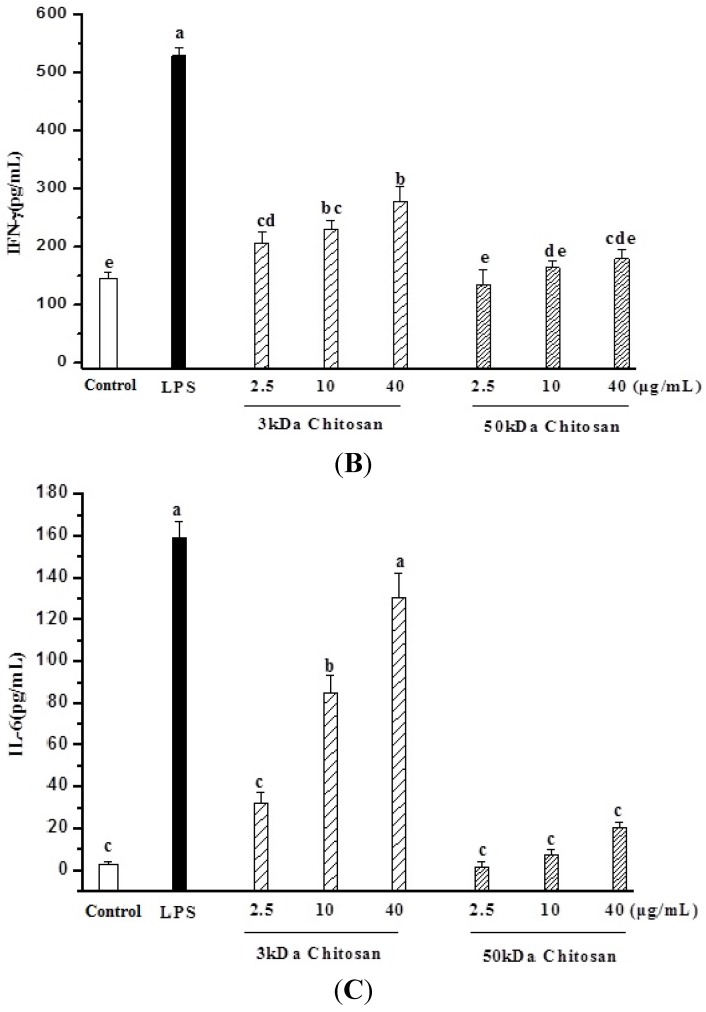
Effects of LMWCs on the production of tumor necrosis factor α (TNF-α) (**A**), interferon-γ (IFN-γ) (**B**) and interleukin 6 (IL-6) (**C**) from RAW264.7 macrophage. Each cell population (1 × 10^6^ cells/mL) was treated with LMWCs (3 kDa and 50 kDa) at the indicated concentrations of 2.5, 10 and 40 μg/mL for 24 h, respectively. Values are means ± SD (*n* = 3). Bars with different letters (a, b, c, d, e, f) are statistically different (*p* < 0.05).

RAW264.7 cells were treated with LMWCs for 24 h, and the secretion levels of TNF-α, INF-γ and IL-6 in the supernatant were detected using ELISA kits. Untreated RAW264.7 cells secrete a basal level of TNF-α and INF-γ but barely detectable amounts of IL-6 (Figure 3). The addition of LMWCs (3 kDa and 50 kDa) resulted in remarked increase in TNF-α secretion levels in a dose-dependent manner (*p* < 0.05) (Figure 3A). Meanwhile, 3 kDa chitosan also induced remarkably increase in INF-γ and IL-6 (*p <* 0.05) but 50 kDa chitosan did not result in that (Figure 3B,C).

### 2.4. Effect of LMWCs on Nitric Oxide (NO) Production and Activities of Inducible Nitric Oxide Synthase (iNOS) in RAW264.7 Macrophage

**Figure 4:**
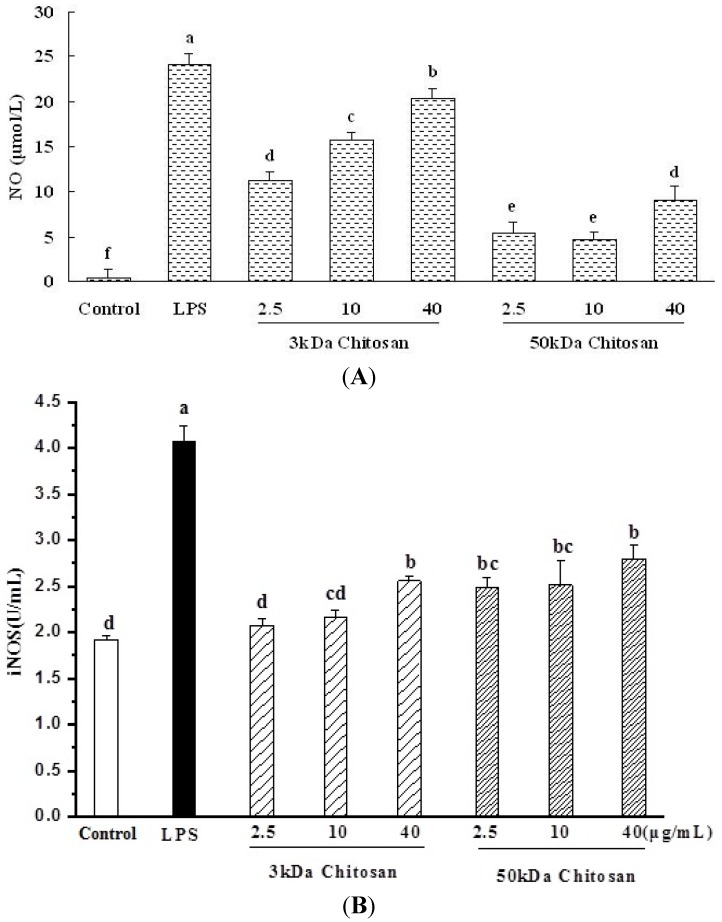
Effect of LMWCs on Nitric oxide (NO) production and activities of inducible nitric oxide synthase (iNOS) in RAW264.7 macrophage. Each cell population (1 × 10^6^ cells/mL) was treated with LMWCs (3 kDa and 50 kDa) at the indicated concentrations of 2.5, 10 and 40 μg/mL for 24 h, respectively. Values are means ± SD (*n* = 3). Bars with different letters (a, b, c, d, e, f) are statistically different (*p* < 0.05).

As shown in Figure 4A, a minimum level of NO was released when RAW264.7 cells were exposed to medium alone, whereas Nitric oxide (NO) production increased in a concentration-dependent. The 3 kDa chitosan induced significantly secretion levels of NO compared with 50 kDa chitosan (*p* < 0.05). Taken together, the results suggested that LMWCs significantly induced the production of NO from RAW264.7 macrophage cells in a molecular weight size and concentration-dependent manner.

RAW264.7 cells were treated with LMWCs for 24 h, and the concentration of inducible nitric oxide synthase (iNOS) in the supernatant was detected using ELISA kit (Figure 4B). The addition of LMWCs resulted in remarkable increase of iNOS secretion levels in a concentration-dependent manner (*p* < 0.05).

### 2.5. Effect of LMWCs on the mRNA Expression Levels of TNF-α and iNOS in RAW264.7 Macrophage

**Figure 5:**
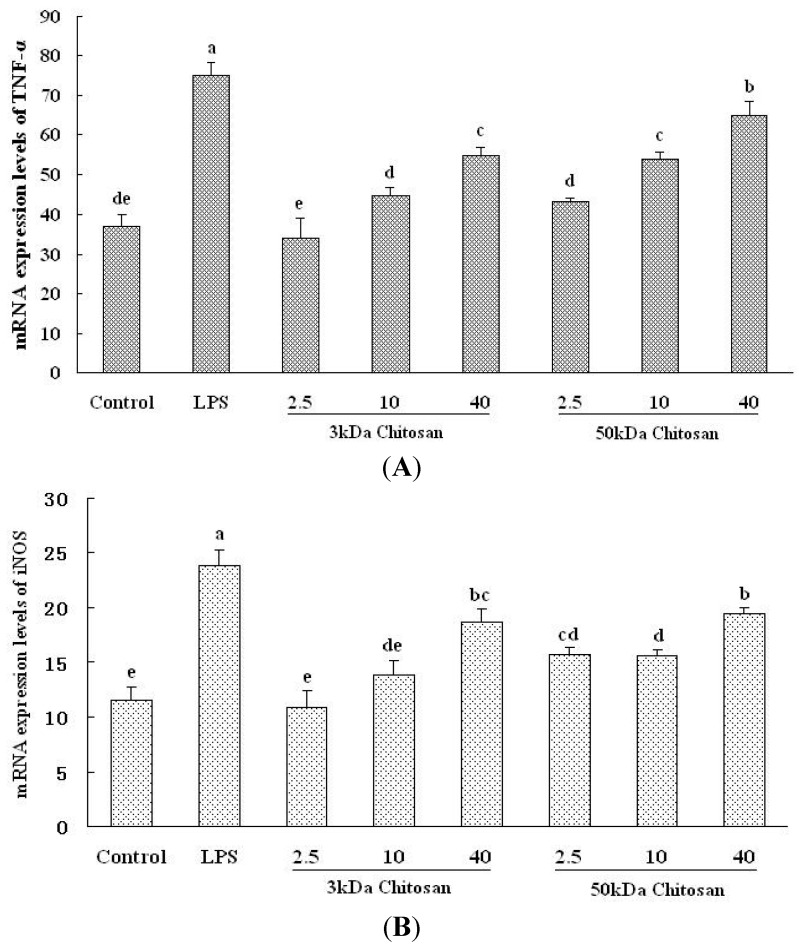
Effect of LMWCs on the mRNA expression levels of TNF-α and iNOS in RAW264.7 macrophage. Each cell population (1 × 10^6^ cells/mL) was treated with LMWCs (3 kDa and 50 kDa) at the indicated concentrations of 2.5, 10 and 40 μg/mL for 24 h, respectively. Values are means ± SD (*n* = 3). Bars with different letters (a, b, c, d, e, f) are statistically different (*p* < 0.05).

Inflammatory factors and its signaling molecules play the prominent role in the maturation and function of macrophages, the potentials for LMWCs to regulate the expression of these mediators in RAW264.7 cells were investigated. As shown in Figure 5A,B, the mRNA expression levels of TNF-α and iNOS in RAW264.7 cells were evaluated by Q-PCR (Real-time Quantitative PCR Detecting System). Under LMWCs stimulation, the mRNA expression levels of TNF-α and iNOS significantly increased in RAW264.7 cells (*p* < 0.05). Both 3 kDa and 50 kDa chitosan significantly induced the mRNA expression levels of TNF-α and iNOS in a concentration-dependent manner (*p* < 0.05).

## 3. Discussion

Owing to the presence of amine, acetylated amine groups and hydroxyl, chitosan and LMWCs interact readily with various cell receptors that triggers a cascade of interconnected reactions in living organisms resulting in anti-inflammatory [13], anti-diabetic [14], anti-microbial [15], anti-HIV-1 (Human Immunodeficiency Virus) [16], anti-oxidant [17], anti-angiogenic [18] and neuroprotective [19] effects. Chitosan and chitin has previously been reported to possess the immunological enhancement as novel adjuvants for vaccines. Chitin has complex and size-dependent effects on innate and adaptive immune responses, which include the ability to recruit and active innate immune cells and induce cytokine and chemokine production [20,21]. However, relationship between molecule weight properties and immunomodulatory activity, and the exact immune regulatory effects of chitosan remain controversial and the mechanisms that mediate these complex effects are still poorly defined.

One of the most distinguished features of macrophage activation would be an increase in pinocytic activity. Therefore, the effects of LMWCs on the pinocytic activity of RAW264.7 cells were determined using neutral red assay. In the previous studies, given by intravenous administration, significant priming effects of chitosan particles in alveolar macrophages in mice was observed, and the phagocytosable small-sized chitosan activated alveolar macrophage to express cytokines such as tumor necrosis factor-α (TNF-α) [21]. In the present study, the results suggested that LMWCs could significantly prime macrophages for an enhanced pinocytic activity in a concentration- and molecular weight-dependent manner. Our results are in agreement with reports in which phagocytosis could play a role in macrophages polarization since chitosan particles stimulated TGF-β1 and PDGF release from *in vitro* cultured macrophages [22].

Macrophages represent a family of mononuclear leukocytes that are widely distributed throughout the body. As the first defense effector of host body, macrophages can recognize the invading microorganisms and tumor cells, as well as eliminate the invaders. Activiated Macrophages can produce many kinds of cytokines, such as TNF-α, IL-1β, IL-6, and IL-10, which are involved in the defense functions and inflammation. Macrophages actively participate in immune responses by releasing proinflammatory cytokines (TNF-α, IFN-γ and IL-6) and inflammatory factors such as NO [23]. The different cytokine production is a key feature of acticated and polarized macrophages. The cytokines associated with T helper cell 1 (Th1) immune response include TNF-α and IFN-γ. In contrast, T helper cell 2 (Th2) cytokines include IL-6. Many reports suggest that chitosan can upregulate TNF-α, IL-1β, and NO production in macrophages [24,25]. However, which types of chitosan activate macrophages that lead to different immunological response is not clear. Several factors, such as the administration route, molecular size and particle size, might account for the Th1 *vs.* Th2 response to chitin [26]. In the previous study, water-soluble, low molecular weight chitosan (MW, 21–92 kDa) had specific immunomodulatory effects on Der f-stimulated human MDM (monocyte-derived macrophages) including the shifting of Th2 cytokine polarization, decreasing the production of the inflammatory cytokines IL-6 and TNF-α [27]. In the present study, 3 kDa chitosan significantly promoted the production of TNF-α, IFN-γ and IL-6 from RAW264.7 cells, moreover, 3 kDa chitosan also up-regulated the mRNA expression levels of TNF-α. All these results suggested that 3 kDa chitosan would simultaneously induced Th1- and Th2-type response; however, 50 kDa chitosan promoted the production of TNF-α, which was consistent with a Th1 response elicited when macrophages phagocytose microparticles of chitin [28].

NO is a free radical formed biologically through the oxidation of l-arginine by nitric oxide synthases. Inducible nitric oxide synthase (iNOS) is key enzyme generating nitric oxide (NO) from the amino acid l-arginine in macrophage cells. iNOS-derived NO plays an important role in numerous physiological (e.g., blood pressure regulation, wound repair and host defense mechanisms) and pathophysiological (inflammation, infection, neoplastic diseases, liver cirrhosis, and diabetes) conditions [29]. It is induced in diseases associated with inflammation and oxidative stress [30]. Previously, LMWC (20 kDa chitosan) inhibited NO production in IFN-γ-induced RAW264.7 cells, whereas the chitooligosaccharides (composed of 1–6 sugars) enhanced NO production [31]. Our results are in agreement with reports in what was previously reported in cultured rat peritoneal macrophages where NO was increased by both low (50 kDa) and high molecular weight chitosan stimulation [32,33]. NO generation by iNOS also influences the cytotoxicity of macrophages and tumor-induced immunosuppression. NO production by high molecular weight water-soluble chitosan (WSC, 300 kDa) indicates that it may provide various activities such as anti-microbial, anti-tumoral, and anti-viral activities under specific conditions *in vivo* [34]. In our study, two LMWCs (3 kDa and 50 kDa) significantly promoted the secretion of NO through up-regulating the mRNA expression levels of iNOS in RAW264.7 cells. The results further indicated that LMWCs effectively activated macrophages in a molecule size- and concentration-dependent manner.

Chitosan has three types of reactive functional groups, an amino/acetamido group as well as both primary and secondary hydroxyl groups at the C-2, C-3 and C-6 positions, respectively. The amino contents are the main factors contributing to the differences in their structures and physicochemical properties. Another important characteristic to consider for chitosan is the molecular weight (MW) or chain length [35]. By modulating and improving physicochemical properties, chitosan and its derivatives may provide novel therapeutic applications for the prevention or treatment of chronic diseases [11]. Following activation, macrophages can induce expression of accessory and costimulatory molecules that promote sustained stimulatory interactions with T cells and the generation of adaptive immunity. Indeed, the basic mechanisms of the immunostimulatory, anti-tumor, bactericidal and other therapeutic effects of polysaccharides are thought to occur via activation of immune cells resulting in the induction of immune responses [36]. Macrophages were thought to be the important target cells of some antitumor and immunomodulatory drug [5]. Stimulation of macrophage response is one of the most important mechanisms of all known polysaccharides with immunological competence. Several pathways are usually involved in this process: (1) improving phagocytic activity; (2) increasing NO and reactive oxygen species (ROS) production; and (3) inducing or regulating the secretion of cytokines and chemokines [37]. In the present study, we proved that LMWCs significantly enhanced the abilities of RAW264.7 macrophages to take up neutral red, produce NO, and induce the secretion of cytokines (TNF-α, IFN-γ and IL-6) and regulate their mRNA expression, which indicated that LMWCs could enhance the immune response via macrophages stimulation in a molecular weight- and concentration-dependent manner. Although both the two LMWCs (3 kDa and 50 kDa) are composed of polymerized glucosamine and *N-*acetyl glucosamine, their molecular sizes are greatly different, with the former (3 kDa) being much smaller than the latter (50 kDa). Accordingly, they may have different bioactivities. The molecular size of chitosanous products seems to be crucial for their immune bioactivity, a similarity shared by some polysaccharides from mushrooms and seaweeds [38,39,40]. Our results showed that the low-MW chitosans (3 kDa) had better immunomodulatory effects than those of higher MW (50 kDa). Zhou *et al.* had reported that λ-carrageenans with MWs of 15 and 9.3 kDa had the best immunomodulatory effects [41]. Lai *et al.* had also reported that low-MW chitosan oligosaccharides had best physiological effects, such as decreasing cholesterol and reinforcing the immune system, which was consistent with our results [42]. LMWCs seem to affect the immune response of macrophages depending upon the molecular weight due to LMWCs with more active amino and hydroxyl groups, but further investigation is necessary to clarify the mechanism of LMWCs.

## 4. Experimental Section

### 4.1. Chemicals and Reagents

Dulbecco’s modified Eagle’s medium (DMEM), penicillin/streptomycin, and the other materials required for culture of cells were purchased from Gibco BRL, Life Technologies (Grand Island, NY, USA). H_2_O_2_, dimethylsulfoxide (DMSO), 3-(4,5-dimethylthiazol-2-yl)-2,5-diphenyltetrazolium bromide (MTT), lipopolysaccharide (LPS), and bovine serum albumin (BSA) were obtained from Sigma (St. Louis, MO, USA). Trizol was from Invitrogen (Carlsbad, CA, USA), revert Aid™ M-MuLV reverse transcriptase was from Fermentas (Amherst, NY, USA), diethylpyrocarbonate (DEPC) and ribonuclease inhibitor were from BioBasic (Markham, ON, Canada), oligo (dT)_18_ were from Sangon (Shanghai, China). All other chemicals were of analytical grade or of the highest grade available commercially.

The LMWCs were sterilized by passing it through a 0.22-µm Millipore filter (Cat No. SLGP033RB, Billerica, MA, USA) to remove any contaminant and then analyzed for endotoxin level by a gel-clot Limulus amebocyte lysate assay (Zhejiang A and C Biological, Zhejiang, China). The concentration of LMWCs was determined by spectrophotometry with bromocresol green according to Zheng *et al*. [43]. The molecular weights of the chitosan were measured using a gel permeation chromatography system (GPC) (Waters 600E, Waters Co., Milford, MA, USA). The molecular weight range of LMWCs: 46 kDa–54 kDa and 2.4 kDa–3.2 kDa. The endotoxin level in the stock soln. was less than 0.5 EU/mL.

### 4.2. Cell Culture and Treatment

Mouse macrophages RAW 264.7 cell line was obtained from the Shanghai Institute of Cell Biology (Shanghai, China) and maintained in DMEM, supplemented with heat-inactivated 10% fetal bovine serum, 100 U/mL penicillin, 100 U/mL streptomycin in a humidified atmosphere of 5% CO_2_ at 37 °C. When the cells reached sub-confluence, they were treated for 24 h with culture medium containing different concentrations of LMWCs, two MW, 3 kDa and 50 kDa (2.5, 10, and 40 μg/mL), lipopolysaccharide (LPS) (1 μg/mL) that were tested in the experiments.

### 4.3. Cell Viability Assay of RAW264.7 Macrophages

The effect of LMWCs on the viability of RAW264.7 macrophages was determined by MTT method. RAW264.7 macrophages were seeded at 2 × 10^4^ cells/well in a 96-well plate and incubated at 37 °C in a humidified atmosphere with 5% CO_2_. After 24 h, the various concentrations of LMWCs were added into each well and these cells were incubated for 24 h. Each concentration was repeated six wells. The cells were washed with PBS and incubated with MTT (5 mg/mL) in culture medium at 37 °C for another 4 h. After MTT removal, the colored formazan was dissolved in 150 μL of DMSO. The absorption values were measured at 490 nm using a SpectraMax M5 Microplate Reader (Molecular Devices, MDS Analytical Technologies, Sunnyvale, CA, USA). The viability of RAW264.7 macrophages in each well was presented as percentage of control cells.

### 4.4. Pinocytic Activity Assay

Pinocytic activity assay was measured as previously [20]. Briefly, RAW264.7 cells were seeded at 1 × 10^4^ cells/well in the 96-well plate and incubated at 37 °C in a humidified atmosphere with 5% CO_2_. After 24 h, DMEM medium, LPS or the various concentrations of LMWCs were added into each well, and these cells were incubated at 37 °C for 24 h. Each concentration was repeated six wells. Culture media were removed and 100 μL/well of 0.075% neutral red was added, and incubated for 30 min. After washed with PBS for three times, 150 μL of cell lyzing solution were added into each well and cells were put at 37 °C for 1 h. The absorbance was evaluated in a SpectraMax M5 Microplate Reader (Molecular Devices, MDS Analytical Technologies, Sunnyvale, CA, USA) at 570 nm.

### 4.5. Preparation of Cell Lysates

The cells were seeded at a density of 1 × 10^6^ cells/mL in 6-well plates. When the cells reached sub-confluence, they were treated for 24 h with culture medium containing different concentrations of LMWCs of two MW, 3 kDa and 50 kDa (2.5, 10, and 40 μg/mL), and LPS (1 μg/mL). Upon completion of the incubation studies, the culture supernatant was collected for analysis of NO release and cytokines. The cells were scraped from the plates into ice-cold 1% Triton X-100 lysis buffer and protein concentration was determined by the bicinchoninic acid (BCA) method, using BSA as a reference standard. Aliquots were stored at −80 °C until detection for the activities of iNOS.

### 4.6. Measurement of Cytokine Levels in RAW264.7 Macrophage Cultures Using an Enzyme Linked Immunosorbent Assay (ELISA)

The RAW264.7 macrophages culture supernatants in each individual treatment were collected to measure pro-inflammatory cytokines TNF-α, IFN-γ and IL-6 levels. The TNF-α, IFN-γ and IL-6 levels were assayed according to the cytokine ELISA protocol from the manufacturer's instructions (Wuhan Boster Biological Engineering Co., Ltd., Wuhan, China).

### 4.7. Measurement of Nitric Oxide (NO) Release and Intracellular Contents of iNOS

The concentration of nitriles (NO_2_^−^) and nitrates (NO_3_^−^), stable end products of nitric oxide (NO), were determined by the reagent kits from the Nanjing Institute of Jiancheng Bioengineering (Nanjing, China). NO production was determined by measuring the optical density at 550 nm and expressed as units per liter. Activity of iNOS in the supernatant was quantified using the iNOS activity assay kit (Nanjing Jiancheng Bioengineering Institute, Nanjing, China) according to the manufacturer’s instructions. Values of iNOS level were expressed as activity units per milligram protein.

### 4.8. Measurement of the mRNA Expression Levels of iNOS and TNF-α by Real-Time PCR

Cells were lysed in 1 mL of Trizol reagent (Invitrogen™, Carlsbad, CA, USA) and the total RNA was isolated according to the manufacture’s protocol. The concentration of total RNA was quantified by determining the optical density at 260 nm. The total RNA was used and reverse transcription (RT) was performed using 1st-Strand cDNA Synthesis Kit (Invitrogen, Waltham, MA, USA). Briefly, nuclease-free water was added giving a final volume of 5 µL after mixing 2 µg of RNA with 0.5 µg oligo (dT)_18_ primer in a DEPC-treated tube. This mixture was incubated at 65 °C for 5 min and chilled on ice for 2 min. Then, a solution containing 3 µL of RT buffer Mix, 0.65 µL of RT Enzyme Mix and 1.35 μL Primer Mix, giving a final volume of 10 µL, and the tubes were incubated for 10 min at 30 °C. The tubes then were incubated for 30 min at 42 °C. Finally, the reaction was stopped by heating at 70 °C for 15 min. The samples were stored at −20 °C until further use.

As shown in Table 1, the primers were used to amplify cDNA fragments (141-bp iNOS fragment, 88-bp TNF-α fragment and 94-bp 18S fragment). Amplification was carried out in total volume of 25 µL containing 1 µL (5 µM) of each target and 18S specific primers, 1 µL of cDNA template, 12.5 µL of Power SYBR^®^ Master Mix (2×) (4 µL of 10× PCR buffer, 4 μL of MgCl_2_ (25 mM), 4 μL of dNTPs (2.5 mM) and 0.5 μL of Taq DNA polymerase) (Invitrogen, Waltham, MA, USA), and 10.5 µL of DEPC-treated water was added. Reaction conditions were the standard conditions for the iQTM5 PCR (Bio-Rad, Hercules, CA, USA) (10 s denaturation at 95 °C, 25 s annealing at 64 °C (TNF-α, iNOS, 18S) with 40 PCR cycles. Ct values were obtained automatically using software (Bio-Rad, Hercules, CA, USA). The comparative Ct method (2^−ΔCt^ method) [44] was used to analyze the expression levels of TNF-α.

**Table 1:** Real-Time Polymerase Chain Reaction (PCR) Primers and Conditions.

Gene	Genbank Accession	Primer Sequence	Product Size (bp)	Annealing (°C)
TNF-α	NM_013693	CGGTGCCTATGTCTCAGCCTCTT	88	64
		GACCGATCACCCCGAAGTTCAGTA		
iNOS	NM_010927.3	TGCCACGGACGAGACGGATA	141	64
		AGGAAGGCAGCGGGCACAT		
18s	NR_003278	CGGACACGGACAGGATTGACA	94	64
		CCAGACAAATCGCTCCACCAACTA		

### 4.9. Statistical Analysis

Data were expressed as mean ± standard deviation (S.D.) and examined for their statistical significance of difference with ANOVA and a Tukey *post hoc* test by using SPSS 16.0. *p*-Values of less than 0.05 were considered statistically significant.

## 5. Conclusions

To the best of our knowledge, this is the first report to demonstrate that two important LMWCs (3 kDa and 50 kDa) can induce activation of RAW264.7 macrophages, which may account for their immune-stimulating effects. We observed that two LMWCs significantly enhance the pinocytic activity, and induce the production of tumor necrosis factor α (TNF-α), interleukin 6 (IL-6), interferon-γ (IFN-γ), nitric oxide (NO) and inducible nitric oxide synthase (iNOS) in a concentration-dependent manner. LMWCs were further shown to upregulate the expression of the genes including iNOS and TNF-α. Moreover, 3 kDa chitosan would simultaneously induced Th1- and Th2-type response, and induced stronger immunostimulative activity than that of 50 kDa chitosan. LMWCs seem to affect the balance of the Th1/Th2 immune response of RAW264.7 cells depending upon the molecular weight due to LMWCs with more active amino and hydroxyl groups. In addition, LMWCs, which are more effectively absorbed in the body than that of high molecular weight chitosan, suitable narrow molecular weight distribution and non-toxicity, could be applied most promisingly to pharmaceutical materials. Accordingly, further investigation is necessary to clarify the mechanism of LMWCs as a novel immunomodulator.
